# Electrical Vestibular Stimuli Evoke Robust Muscle Activity in Deep and Superficial Neck Muscles in Humans

**DOI:** 10.3389/fneur.2018.00535

**Published:** 2018-07-05

**Authors:** Patrick A. Forbes, Jason B. Fice, Gunter P. Siegmund, Jean-Sébastien Blouin

**Affiliations:** ^1^Department of Neuroscience, Erasmus Medical Centre, Rotterdam, Netherlands; ^2^Department of Biomechanical Engineering, Faculty of Mechanical, Maritime and Materials Engineering, Delft University of Technology, Delft, Netherlands; ^3^School of Kinesiology, University of British Columbia, Vancouver, BC, Canada; ^4^MEA Forensic Engineers & Scientists, Richmond, BC, Canada; ^5^Djavad Mowafaghian Centre for Brain Health, University of British Columbia, Vancouver, BC, Canada; ^6^Institute for Computing, Information and Cognitive Systems, University of British Columbia, Vancouver, BC, Canada

**Keywords:** cervical vestibular-evoked myogenic potentials, deep and superficial neck muscles, electrical vestibular stimulation, vestibulocollic pathways, isometric neck muscle contractions

## Abstract

Neck muscle activity evoked by vestibular stimuli is a clinical measure for evaluating the function of the vestibular apparatus. Cervical vestibular-evoked myogenic potentials (cVEMP) are most commonly measured in the sternocleidomastoid muscle (and more recently the splenius capitis muscle) in response to air-conducted sound, bone-conducted vibration or electrical vestibular stimuli. It is currently unknown, however, whether and how other neck muscles respond to vestibular stimuli. Here we measured activity bilaterally in the sternocleidomastoid, splenius capitis, sternohyoid, semispinalis capitis, multifidus, rectus capitis posterior, and obliquus capitis inferior using indwelling electrodes in two subjects exposed to binaural bipolar electrical vestibular stimuli. All recorded neck muscles responded to the electrical vestibular stimuli (0–100 Hz) provided they were active. Furthermore, the evoked responses were inverted on either side of the neck, consistent with a coordinated contribution of all left-right muscle pairs acting as antagonists in response to the electrically-evoked vestibular error of head motion. Overall, our results suggest that, as previously observed in cat neck muscles, broad connections exist between the human vestibular system and neck motoneurons and highlight the need for future investigations to establish their neural connections.

## Introduction

Vestibular-evoked myogenic potentials measured in cervical muscles (i.e., cVEMPs) are commonly used to assess vestibular function. The evoked muscle activity is characterized by a short-latency vestibular response to air-conducted sound (clicks), bone-conducted vibration or electrical vestibular stimulation. cVEMPs are typically measured in the sternocleidomastoid (SCM) muscle using surface electrodes. The evoked muscle response is a short-latency biphasic waveform that is produced by the inhibition (or excitation) of individual motor units (1). As a result, background muscle activity is required to measure a reliable response, and can be achieved by asking subjects to raise their head from a supine position and/or turn their head away from the recorded SCM muscle. The relative ease in evoking and recording cVEMPs in SCM has led to its extensive use in the clinic, and forms part of neuro-otological diagnostic tests for both peripheral (primarily otolithic) vestibular and central nervous system disorders (2).

Biphasic responses to vestibular stimuli have also been reported in the dorsally located splenius capitis (SPL) muscle (3–9). The initial response in SPL recordings, much like the SCM muscle, is associated with a decrease in multi-unit activity (9). The response similarities in contralateral SCM and SPL align with the agonist recruitment of these two muscles for head turns, and are thought to reflect the synergistic activity by descending vestibulospinal neurons (9). Synergies for these two muscles, however, are not fixed: they act as agonists during head turns and antagonists during flexion or extension. To achieve this flexibility, single vestibulospinal (and reticulospinal) neurons branch to multiple combinations (i.e., synergies) of neck motoneurons (10–13). Therefore, it is unlikely that response similarities between two muscles are due to single descending agonist activation synergies, since fixed vestibulospinal relationships would not lend themselves well to the flexible control of neck muscles. In support of this proposition, changes in descending motor commands in humans do not modify the vestibular-evoked reflex in neck muscles (8). Given the widespread connections of both vestibular end organs (canal and otolith) to all neck motoneurons in cats [see reviews by (14) and (12)], it is possible that all human neck muscles respond to vestibular activity and that any neck muscle could be used to measure cVEMPs. It is currently unknown, however, whether human neck muscles other than SCM and SPL respond to vestibular input. To examine this question, we recorded vestibulocollic reflexes evoked by electrical vestibular stimulation in seven bilateral deep and superficial neck muscles in two human subjects during isometric neck muscle contractions in axial rotation, flexion and extension.

## Methods

### Subjects

Two healthy male subjects [age 36 and 29 years, height 180 and 178 cm, weight 78 and 76 kg, respectively] with no self-reported history of neurological disorders participated in this study. Both subjects were co-investigators (PAF, JBF). The protocol was explained prior to the experiment and both subjects gave their written informed consent. The experiment conformed to the Declaration of Helsinki and was approved by the University of British Columbia's Clinical Research Ethics Board.

### Vestibular stimuli

Subjects were exposed to a binaural-bipolar electrical vestibular stimulation (EVS) delivered over the mastoid processes behind both ears using carbon rubber electrodes (~9 cm^2^). The electrodes were coated with Spectra 360 electrode gel (Parker Laboratories, Fairfield, NJ, USA) and secured to the head with hypoallergenic tape (Durapore Surgical Tape, 3M, Maplewood, MN, USA). The stimulus was delivered as an analog signal via a data acquisition board (PXI-6289; National Instruments, Austin, TX, USA) to an isolated constant current stimulator (STMISOL; Biopac, Goleta, CA, USA). Both subjects were exposed to the same stimulus: a 50 s filtered white-noise stochastic vestibular stimulation having a bandwidth of 0–100 Hz and a root-mean-square (RMS) current of 1.71 mA (amplitude peak ± 4 mA) (6). By convention, the vestibular signal was positive for anode right/cathode left currents and negative for cathode right/anode left currents. Binaural-bipolar EVS modulates the firing rate of canal and otolith primary afferents bilaterally, decreasing firing rates on the anode side and increasing firing rates on the cathode side (15, 16). The net afferent activity evokes a vestibular error signal that is perceived as a sensation of head rotation about a roll axis fixed in head coordinates (17). The signals were generated offline using Matlab software (Mathworks, Natick, MA, USA), and identical signals were delivered to each subject.

Short-duration (2 ms) square-wave pulses are more commonly used to produce electrically-evoked cVEMPs. The use of stochastic stimulation has received recent attention due to the advantages it brings over square-wave stimuli. First, detailed information about the muscular responses to the electrical stimulation can be obtained at all frequencies included in the stimulus (6, 18, 19). In turn, cross-correlation between the stimulus and muscle activity is equivalent to responses evoked by square-wave stimuli (8, 20). Furthermore, stochastic stimuli offer several experimental advantages, including increased signal-to-noise ratios (21, 22), minimized anticipation to the stimulus (23), reduced experimental durations (20), and less irritation or nausea evoked by the stimulus (20).

### Instrumentation

Intramuscular electromyography (EMG) was recorded bilaterally in the sternohyoid (STH), sternocleidomastoid (SCM), splenius capitis (SPL), semispinalis capitis (SCP), multifidus (MULT), rectus capitis posterior (RCP) and obliquus capitis inferior (OCI) using indwelling electrodes. Pairs of 0.05 mm wire (Stablohm 800A; California Wire, Grover Beach, CA, USA) were inserted under ultrasound guidance (Mircormaxx; Sonosite, Bothell, WA, USA). One of the two wires from each electrode had 2–3 mm of exposed wire to allow for recording of multi-unit EMG potentials. Wire insertions for the RCP and OCI muscles were placed at the C_1_/C_2_ level, for the SCM, SPL, SCP, and MULT at the C_4_/C_5_ level, and for the STH at the C5/C6 level. All wires were placed near the center of the horizontal cross section of the muscle. In the SCM, the wire always remained superficial to the readily identifiable cleidomastoid subvolume (24). Identification of the suboccipital muscles followed the approach outlined by Cho et al. (25). Briefly, the ultrasound probe was placed on the dorsal side of the neck, lateral from the midline and oriented along a line formed between the palpated spinous process of C2 and transverse process of C1. It was then rotated 90° to view the cross sections of the suboccipital muscles. The insertions of all electrodes were completed over a period of ~1.5 h. All EMG signals were amplified (#x000D7;200–500; Neurolog, Digitimer, Welwyn Garden City, UK) and bandpass filtered (10–2,000 Hz) before digitization. Isometric neck forces and moments were measured with an overhead six-axis load cell (JR3 E-Series, JR3, Woodland, CA, USA). EMG, forces, moment and vestibular stimuli signals were recorded at 10,000 Hz via digital acquisition boards (PXI-4495 & PXI-6289, National Instruments, TX, USA) using a custom LabVIEW software program (National Instruments, TX, USA).

### Protocol

Subjects sat with their torso firmly strapped to a rigid vertical seatback and performed isometric neck muscle contractions with their head clamped to the overhead load cell via a helmet (Pro-Tec, Vans, Cypress, CA). The head was fixed throughout the experiment, facing forward and oriented with the Reid's plane tilted chin up by 18°; this head position maximizes the perception of roll evoked by the electrical stimulus (17, 26). Once secured, subjects practiced contracting their neck muscles in four different isometric contraction directions generating a leftward yaw moment, a rightward yaw moment, a flexion moment, and an extension moment. To control flexion and extension moments generated by the participants, we used anterior and posterior forces measured at the load cell location. These four arrangements of isometric contraction were chosen to ensure that all muscles were active in at least one of the contraction directions since muscle activity is required to measure electrically evoked vestibulocollic reflexes (2, 8, 27). The practice sessions were also used to establish a suitable target load level (measured moment or force) for the subsequent stimulation trials that would ensure continuous neck muscle activity throughout the trial while avoiding effects of fatigue. Moment and force targets were set at ~10–15 and ~20–25% of expected maximal voluntary contraction values (28, 29) for subject 1 and 2 respectively. Subjects then performed the four isometric voluntary contraction trials (~50 s each) with their eyes closed while being exposed to electrical vestibular stimulation. Subjects were given verbal instructions to maintain the target moment or force while minimizing force/moments along other axes. Each contraction direction was performed twice and the order of the different contraction directions was randomized for each subject. The experimental protocol lasted about 1 h.

### Signal analysis

EMG data were first high-pass filtered with a phaseless 8th-order Butterworth digital filter (−3 dB at 110 Hz) to remove the electrical stimulation artifact (6). Repeated trials within each subject were concatenated to create 100-s data records and analyzed on a subject-by-subject basis. Cumulant density estimates were calculated to evaluate the correlation between the input electrical stimulus and the rectified EMG in the time-domain (30). Cumulant density estimates were derived by taking the inverse Fourier transform of the cross-spectrum (31) and then normalized (between −1 and +1) by the product of the vector norms of the input and output signals (19). In accordance with the stimulus sign convention (see above), a positive cumulant density indicates that an anode right/cathode left current induced an excitation of the muscle activity and an anode left/cathode right current induced an inhibition. In sternocleidomastoid and splenius capitis neck muscles, cumulant density estimates are similar to reflexes evoked by square-wave stimuli and are characterized as short-latency biphasic waveforms with peaks occurring at 10–15 ms and 21–25 ms (8, 32). They are also inverted in bilateral SCM and SPL muscle pairs (8) likely owing to the antagonistic function of the muscles in response to the vestibular error signal of mediolateral head rotation evoked by the electrical stimulus when the head is oriented upright (17, 26). For all muscles in each contraction direction, a normalized 95% confidence interval was calculated to indicate where cumulant density responses were significant (31).

Given the capacity for neck muscles to generate multidirectional forces and movements, we expected that the activity in each muscle would vary across our multiple contraction directions. However, because the amplitude of the electrically-evoked vestibulocollic scales with the level of activity, is absent when the muscle is quiescent (8, 32) and does not vary with descending motor command (8), we limited our analysis for each muscle to the contraction direction with the highest muscle activity. This condition was identified in each muscle using the root-mean-square (RMS) of the filtered EMG. For these specific recordings, a muscle was considered to respond to the electrical stimulus when the cumulant density contained positive or negative peaks correlating with the stimulus (i.e., exceeding the 95% confidence interval) over a lag of 5–30 ms. Based on the extensive projection of vestibular afferents to neck muscles in cat, we expected that all neck muscles would exhibit significant vestibular-evoked muscle (i.e., cumulant density) responses. Furthermore, because the electrical stimulus evokes sensations of head roll motion in the mediolateral direction with the head oriented upright, we expected to observe an inversion in the polarity of the evoked responses across left and right muscle pairs. Finally, we extracted the timing of the cumulant density peaks. Given that vestibulocollic pathways are formed by either disynaptic or trisynaptic connections, we expected that peak response times would occur within the 5–30 ms lag.

## Results

### Neck muscle activity

**Figure 1:**
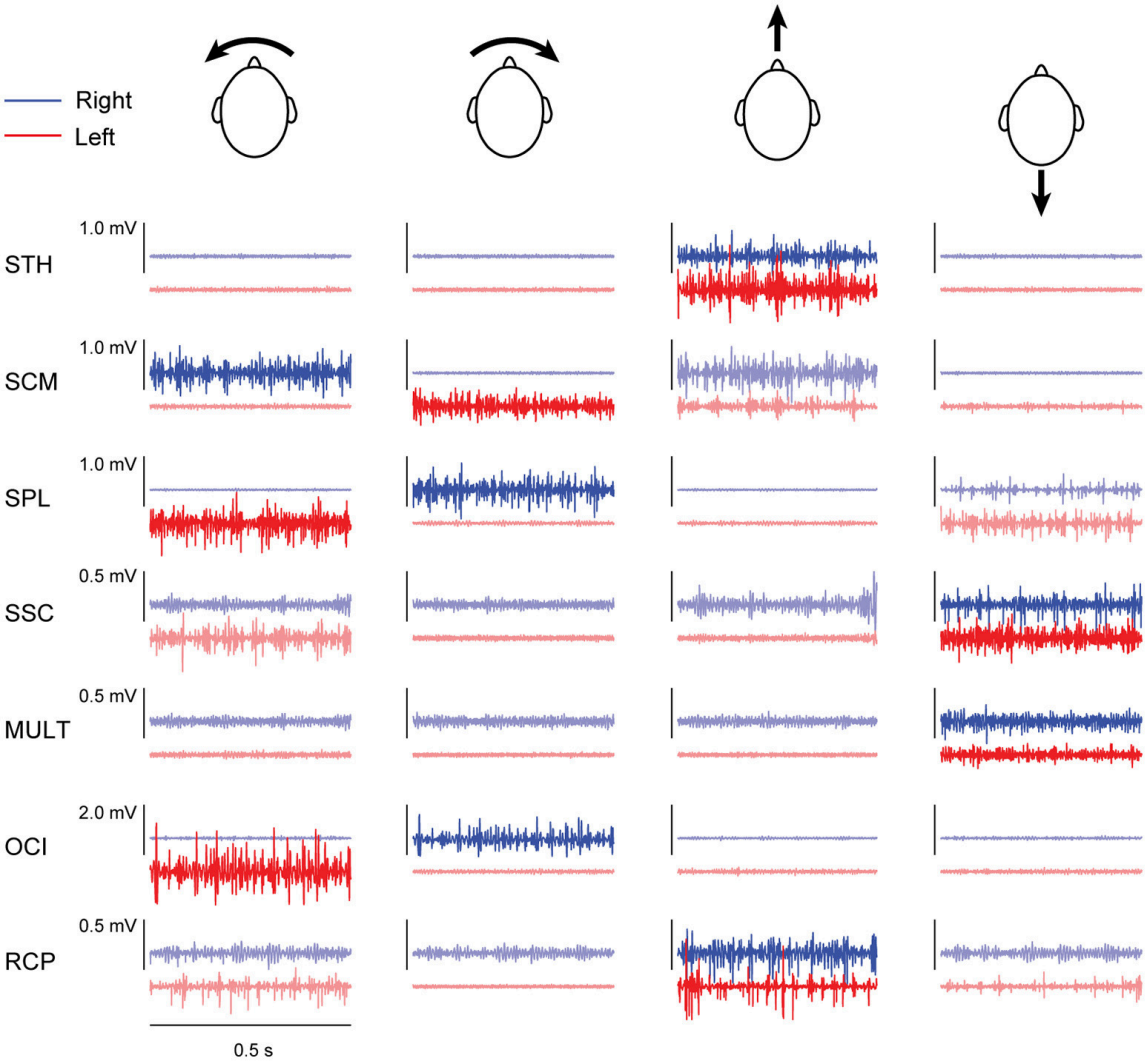
Filtered muscle activity (blue: right muscles; red: left muscles) from subject 2 recorded over 0.5 s within each of the four different contraction directions. Darker lines represent the condition during which muscle activity was used for estimating cumulant density responses. Arrows accompanying the heads indicate the direction of load applied by the head in each contraction direction (columns from left-to-right: leftward yaw moment, rightward yaw moment, flexion moment and extension moment).

Both subjects exhibited patterns of neck muscle activity that depended upon the moment or force direction of each isometric task. Yaw moments were generated by agonist activity of contralateral SCM and ipsilateral SPL muscles, as well as ipsilateral activity of suboccipital muscles RCP and OCI (see Figure 1). Flexion forces were generated by bilateral activity of neck flexor muscles STH and SCM, while extension forces were generated by bilateral activity of neck extensor muscles SPL, SSC and MULT. Although we expected bilateral activity of both suboccipital muscles (RCP and OCI) when generating horizontal extension forces, this was only observed in subject 1 (data not shown). For subject 2, the largest activity in bilateral RCP muscles was produced when generating neck flexion forces (see Figure 1).

### Vestibulocollic reflexes (cVEMPs) are evoked in all muscles

**Figure 2:**
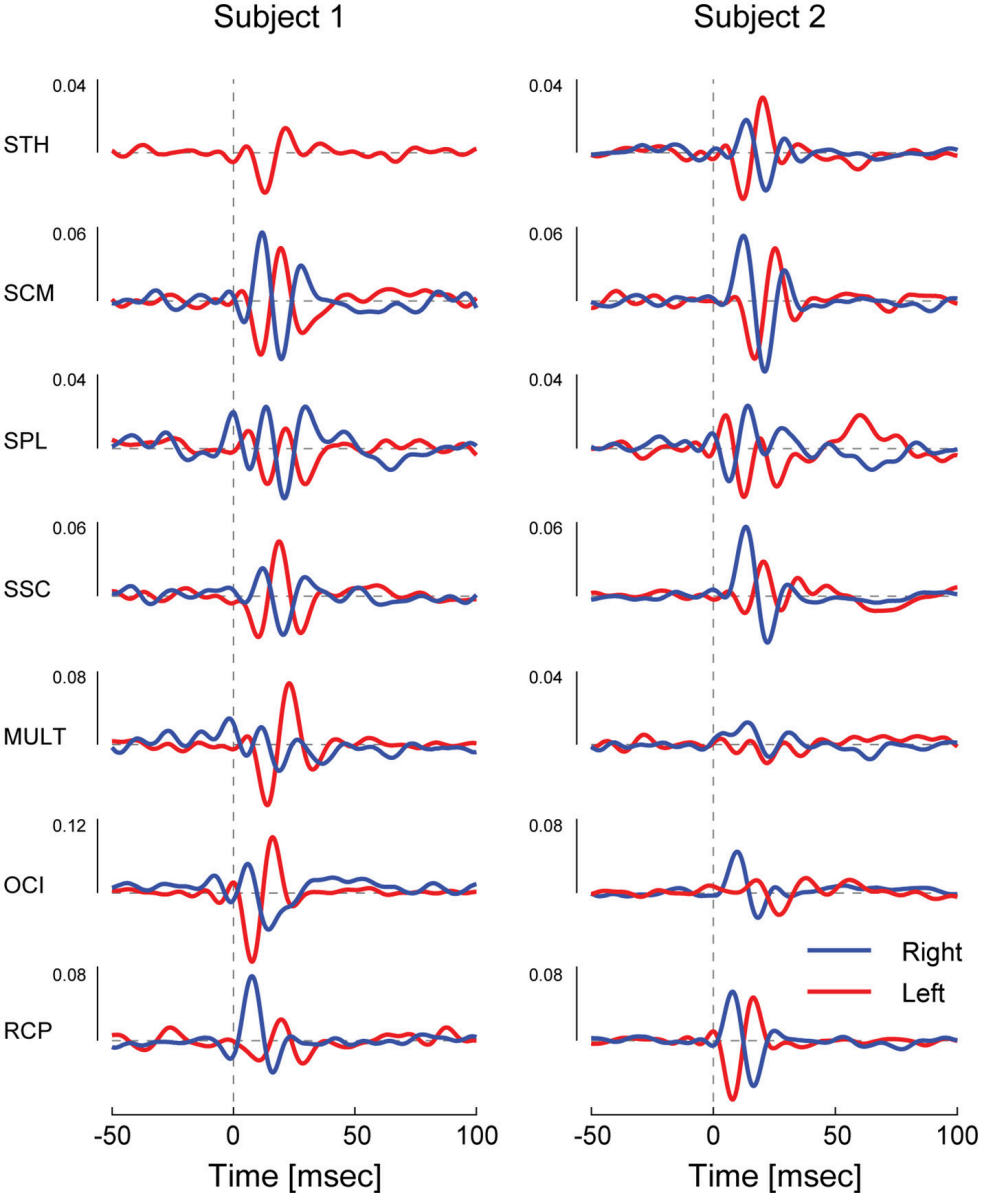
Cervical vestibular-evoked myogenic potentials from both subject in all muscles as estimated using cumulant density responses. In most muscles, the profile of vestibular-evoked responses exhibited a biphasic waveform that was inverted across bilateral muscles pairs. For small cumulant density responses (see subject 2 SPL, SSC, and MULT), spurious oscillations were observed before and after the typical biphasic peak due to low muscle activity (see Figure 1).

Correlation between the electrical stimulus and neck muscle activity exceeded the 95% confidence interval in 27/28 measured neck muscles across both subjects (see Figure 2). In most muscles, the profile of vestibular-evoked responses exhibited a biphasic waveform with peaks occurring at ~6–17 ms (first peak) and ~16–27 ms (second peak: see Table 1). No significant response was evoked in the STH muscle for subject 2 because it remained inactive across all contraction directions. When averaged across subjects (*n* = 2) and muscles (*n* = 14) the first and second peaks occurred at 12.1 ± 2.8 and 20.9 ± 3.2 ms, respectively. These response latencies are consistent with the transmission of the descending vestibular signals over short latency disynaptic or trisynaptic pathways when considering the pathway length and conduction velocity, as shown by animal studies (13, 14). In bilateral muscle pairs, cumulant density responses were inverted, with right-sided muscles exhibiting positive-negative polarities and left-sided muscles showing negative-positive polarities. Some exceptions to these general features were observed in subject 2: SPL, SSC and MULT muscles, where responses were small relative to the surrounding oscillations within the cumulant density function. Spurious oscillations before or after the typical biphasic peaks have been reported previously (8). These occur when muscle activity is low as observed within these muscles in all trials (see Figure 1). Overall, these results indicate a mirrored bilateral response to the input vestibular stimulus with peak timing that is consistent with the short latency pathways that contribute to the vestibulocollic reflexes.

**Table 1:** Latencies of the first and second peaks of the cumulant density estimates of all muscles in both subjects.

**Muscle**	**Subject 1**				**Subject 2**			
	**Right muscles**		**Left muscles**		**Right muscles**		**Left muscles**	
	**First peak**	**Second peak**	**First peak**	**Second peak**	**First peak**	**Second peak**	**First peak**	**Second peak**
STH	NaN	NaN	13.0	21.5	13.6	21.6	12.3	20.3
SCM	11.9	19.8	11.3	19.5	12.5	21.2	17.0	25.5
SPL	13.4	21.0	14.0	21.5	14.2	28.1	12.7	26.1
SSC	12.1	20.5	10.3	18.9	13.5	22.3	13.0	20.7
MUL	11.5	18.8	14.0	23.0	14.2	23.1	15.1	22.1
OCI	6.2	16.0	7.7	16.2	12.0	20.6	17.8	27.1
RCP	7.7	16.3	11.0	19.7	8.0	16.6	8.0	16.5

*In all muscles, peak responses were observed within the 5–30 ms window expected for disynaptic and trisynaptic pathways of vestibulocollic reflexes. Values are in milliseconds*.

## Discussion

The primary aim of this study was to determine whether vestibular-evoked myogenic potentials are evoked in deep and superficial neck muscles using a binaural-bipolar electrical vestibular stimulation applied over the mastoid processes. Our results demonstrate that all measured neck muscles exhibit coupling with the input stimulus given the muscle is active. The presence of electrically activated VEMPs in all measured neck muscles is consistent with the complex and widespread neural connectivity between the vestibular system and neck motoneurons observed in cats (13, 14). Typical neck motoneurons receive inputs from all six semicircular canals and four otolith organs through excitatory and/or inhibitory pathways. The pattern of these connections to each muscle is consistent with a given muscle's function in responding to input from each vestibular end organ (12). For example, anterior and posterior canals, which are active during ipsilateral head roll, produce contralateral excitation and ipsilateral inhibition in lateral flexor muscles. These muscle-specific connections, however, are not exclusive: divergent pathways originating from one vestibular end organ branch to multiple neck motoneurons, while convergent pathways combine afferent signals from multiple end organs prior to termination in the spinal cord (10, 11). In addition, bulbospinal pathways, reticulospinal pathways (33–35) and input from the interstial nucleus of Cajal (36) are known to contribute to the vestibulocollic reflex. Therefore, identifying the specific contributions made by the different end organs or descending pathways within each muscle is difficult based on our observations. Nevertheless, given the functional diversity of these connections, it is plausible that varying combinations of pathways and end-organs contribute to the neck muscle responses observed here, particularly when considering the afferent activity evoked by the vestibular stimulus used here.

Electrical vestibular stimulation, when delivered in a binaural bipolar configuration, modulates the firing rate of both canal and otolith afferents bilaterally (15, 16). Based on the morphology of the vestibular system, the vector sum of this afferent activity is estimated to induce primarily a net signal of angular head roll about an axis directed posteriorly and superiorly by 18° relative to the Reid's plane (17, 26). With the EVS-evoked roll vector aligned with gravity (i.e., when looking toward the floor), this isolated vestibular error signal evokes a virtual sensation of head rotational velocity in the horizontal plane (37). Therefore, despite the aforementioned difficulties in identifying specific contributions, the reversal of cumulant density functions in all bilateral muscle pairs [seen previously in SCM and SPL muscles; (8)] indicates an antagonistic response of left-right muscle pairs to the vestibular error-signal of head motion. Accordingly, neck muscles are capable of generating both laterally directed isometric neck moments (38) and head stabilization during laterally directed torso movements (39), though equivalent loading direction properties have yet to be established with RCP and OCI muscles.

Oppositely-directed vestibular-evoked responses are also observed in bilateral lower-limb muscles when subjects stand with the head facing forward. Vestibular-evoked responses for standing balance, however, appear to be more flexibly organized than equivalent responses for head-neck control. Lower-limb muscles compensate only for the component of the net vestibular-error that is aligned with and thus relevant to the ongoing balance task, and are unresponsive when subjects are fully supported (40–42). More notably, responses in soleus muscle are inverted when the relationship between balancing motor commands and vestibular feedback are reversed (42). Neck muscles in contrast, respond to the stimulus even with the head fixed (8). The flexible organization of vestibular-evoked balance responses for standing are thought to reflect the central processing involved in compensating for the relevant component of the vestibular-error (42, 43), which may be absent (or at least limited) when generating vestibulocollic reflexes for head-neck control. Under this latter assumption, an alternative possibility is that the neck muscle responses to the vestibular-error observed here are simply due to the neural circuitry underlying functional synergies for the control of neck muscles as proposed in cats (13). This is supported by the relative insensitivity of vestibular-evoked neck muscle activity in cats across 25 degree pitch rotations (44) and in humans across 60° yaw rotations (6, 8). Admittedly, however, because neck muscle origin and/or insertion points rotate with the head and neck, the muscle may maintain a similar line of action across different head orientations in response to equivalent vestibular disturbances (45). Therefore, further experiments are needed to test this hypothesis.

From a clinical standpoint, our results support previous suggestions that neck muscles other than SCM could be used as a complementary measure to assess vestibular function (5, 7, 9). For example, electrically-evoked cVEMPs from multiple neck muscles could complement the assessment of age-related decline in either central or peripheral vestibular function (46–48). Alternatively, vestibular stimuli which isolate end-organ activity through natural rotational or translational motion (49) could assess disruptions in organ-specific pathways contributing to each muscle's response. Considering the head motion equivalent of a 1 mA stimulus is ~1–6°/s (37, 51–52), our results indicate that only a small head motion (~4–24°/s) would be required to evoke neck muscle responses of a similar magnitude to those evoked by our electrical stimulus. A similar argument could be made for unilateral air-conducted short-tone bursts, where extensor muscle activity could be used to assess utricle function; which based on cat studies, should receive ipsilateral inhibitory and contralateral excitatory descending input (53, 54). We note, however, that substantial developmental work in healthy controls is required to introduce these techniques in clinical practice, particularly in assessing whether the specific neural pathways making up these circuits match those identified in animals (12–14). Based on the current results that all measured neck muscles respond to vestibular input, additional work can be implemented to examine responses in a larger subject group and under a variety of vestibular stimuli. We also acknowledge the additional load that may be placed on patients in measuring deep neck muscles with indwelling electrodes in the clinic, which is necessary to avoid cross-talk between neck muscles common with surface recordings.

In conclusion, we have shown that cVEMPs can be evoked by electrical stimuli in deep and superficial human neck muscles consistent with the widespread innervation of vestibulospinal neurons previously deduced from cat neck muscles. These results imply that any human neck muscle could be used to measure cVEMPs.

## Data availability statement

The datasets for this manuscript are not publicly available because permission to release them was not obtained from the University of British Columbia's Clinical Research Ethics Board at the time of protocol approval.

## Author contributions

PF, JF, GS, and J-SB contributed to conception and design of the study; GS and J-SB performed the electrode insertions; PF and JF collected the data. PF analyzed the data and wrote the first draft of the manuscript. All authors contributed to manuscript revisions, and read and approved the submitted version.

### Conflict of interest statement

GS owns shares in a consulting company, and both he and the company may derive benefit from being associated with this work. The remaining authors declare that the research was conducted in the absence of any commercial or financial relationships that could be construed as a potential conflict of interest.
